# Comparison of single cell sequencing data between two whole genome amplification methods on two sequencing platforms

**DOI:** 10.1038/s41598-018-23325-2

**Published:** 2018-03-21

**Authors:** DaYang Chen, HeFu Zhen, Yong Qiu, Ping Liu, Peng Zeng, Jun Xia, QianYu Shi, Lin Xie, Zhu Zhu, Ya Gao, GuoDong Huang, Jian Wang, HuanMing Yang, Fang Chen

**Affiliations:** 1 BGI Education Center, University of Chinese Academy of Sciences, Shenzhen, 518083 China; 2 Laboratory of Genomics and Molecular Biomedicine, Department of Biology, University of Copenhagen, DK-2100 Copenhagen, Denmark; 3 School of Bioscience and Bioengineering, South China University of Technology, Guangzhou, 510006 China; 4 BGI-Shenzhen, Shenzhen, China; 5 James D. Watson Institute of Genome Sciences, Hangzhou, 310058 China

## Abstract

Research based on a strategy of single-cell low-coverage whole genome sequencing (SLWGS) has enabled better reproducibility and accuracy for detection of copy number variations (CNVs). The whole genome amplification (WGA) method and sequencing platform are critical factors for successful SLWGS (<0.1 × coverage). In this study, we compared single cell and multiple cells sequencing data produced by the HiSeq2000 and Ion Proton platforms using two WGA kits and then comprehensively evaluated the GC-bias, reproducibility, uniformity and CNV detection among different experimental combinations. Our analysis demonstrated that the PicoPLEX WGA Kit resulted in higher reproducibility, lower sequencing error frequency but more GC-bias than the GenomePlex Single Cell WGA Kit (WGA4 kit) independent of the cell number on the HiSeq2000 platform. While on the Ion Proton platform, the WGA4 kit (both single cell and multiple cells) had higher uniformity and less GC-bias but lower reproducibility than those of the PicoPLEX WGA Kit. Moreover, on these two sequencing platforms, depending on cell number, the performance of the two WGA kits was different for both sensitivity and specificity on CNV detection. The results can help researchers who plan to use SLWGS on single or multiple cells to select appropriate experimental conditions for their applications.

## Introduction

A strategy of single-cell low-coverage whole genome sequencing (SLWGS) is suited for the detection of chromosomal aberrations^1^. Typically, next-generation sequencing (NGS) requires nanogram amounts of DNA to construct a library for sequencing^2^, whereas a single cell only contains 6–7 pg of genomic DNA (gDNA). Therefore, a critical step for single-cell sequencing is whole-genome amplification (WGA) to generate sufficient DNA for library construction.

Three WGA methods are widely used for SLWGS, namely, degenerate-oligonucleotide-primed polymerase chain reaction (DOP-PCR) (marketed as WGA4 kit; Sigma-Aldrich, St. Louis, MO, US)^2^, multiple displacement amplification (MDA) (marketed as REPLI-g Single Cell Kit; QIAGEN, Germantown, MD, US)^3^, and a combination of displacement pre-amplification and PCR amplification (marketed as PicoPLEX WGA Kit; Rubicon Genomics, Ann Arbor, MI, US)^4^. Many comparisons have evaluated the efficiency among these WGA kits^5,6^, and each kit has unique pros and cons. Hou *et al*.^5^ found that DOP-PCR had the highest duplication rate, an even read distribution, and the best reproducibility and accuracy for detection of copy number variations (CNVs) by SLWGS. Huang *et al*.^6^ compared five commercial WGA kits comprehensively by performing deep sequencing of multiple cells and reported that WGA4 kit and PicoPLEX WGA Kit presented the highest reproducibility, with similar coefficients of variation appropriate for accurate detection of CNVs. Ning *et al*.^1^ suggested that the WGA4 kit presented a high level of uniformity that was key to successfully identify CNVs using SLWGS. Generally, the WGA4 kit and the PicoPLEX WGA Kit are widely used WGA methods in SLWGS for detection of CNVs^1–6^. Therefore, their performances must be compared on different sequencing platforms and with different cell numbers to help researchers make the correct choice depending on specific conditions.

Recently, two sequencing platforms have been used extensively in human genome sequencing research. The HiSeq2000 (Illumina, San Diego, CA, US) exploits highly parallel optical sensing of polymerization reactions and sequencing-by-synthesis technology to implement ultra-high throughput sequencing, although the procedure requires long turnaround time (TAT)^7^. For the other platform, Life Technologies released an integrated semiconductor sequencing device, Ion Proton (Thermo Fisher Scientific, Waltham, MA, USA), which is reported to provide shorter TAT and a more cost-effective NGS solution than those of alternative platforms^8^.

In this study, we compared the performance of SLWGS on two platforms (HiSeq2000 and Ion Proton platforms) with two commercial kits (WGA4 kit from Sigma-Aldrich and PicoPLEX WGA Kit from Rubicon Genomics) using the same sample set. We systematically evaluated the performance of four combinations: Rubicon PicoPLEX WGA Kit and single cell (RS), Rubicon PicoPLEX WGA Kit and multiple cells (RM), Sigma-Aldrich WGA4 kit and single cell (SS), Sigma-Aldrich WGA4 kit and multiple cells (SM). Our study showed the strengths and limitations of each combination on the two sequencing platforms, which will provide useful information for choosing the appropriate WGA kit when confronted with different cell numbers and sequencing platforms.

## Methods

### Design overview

**Figure 1:**
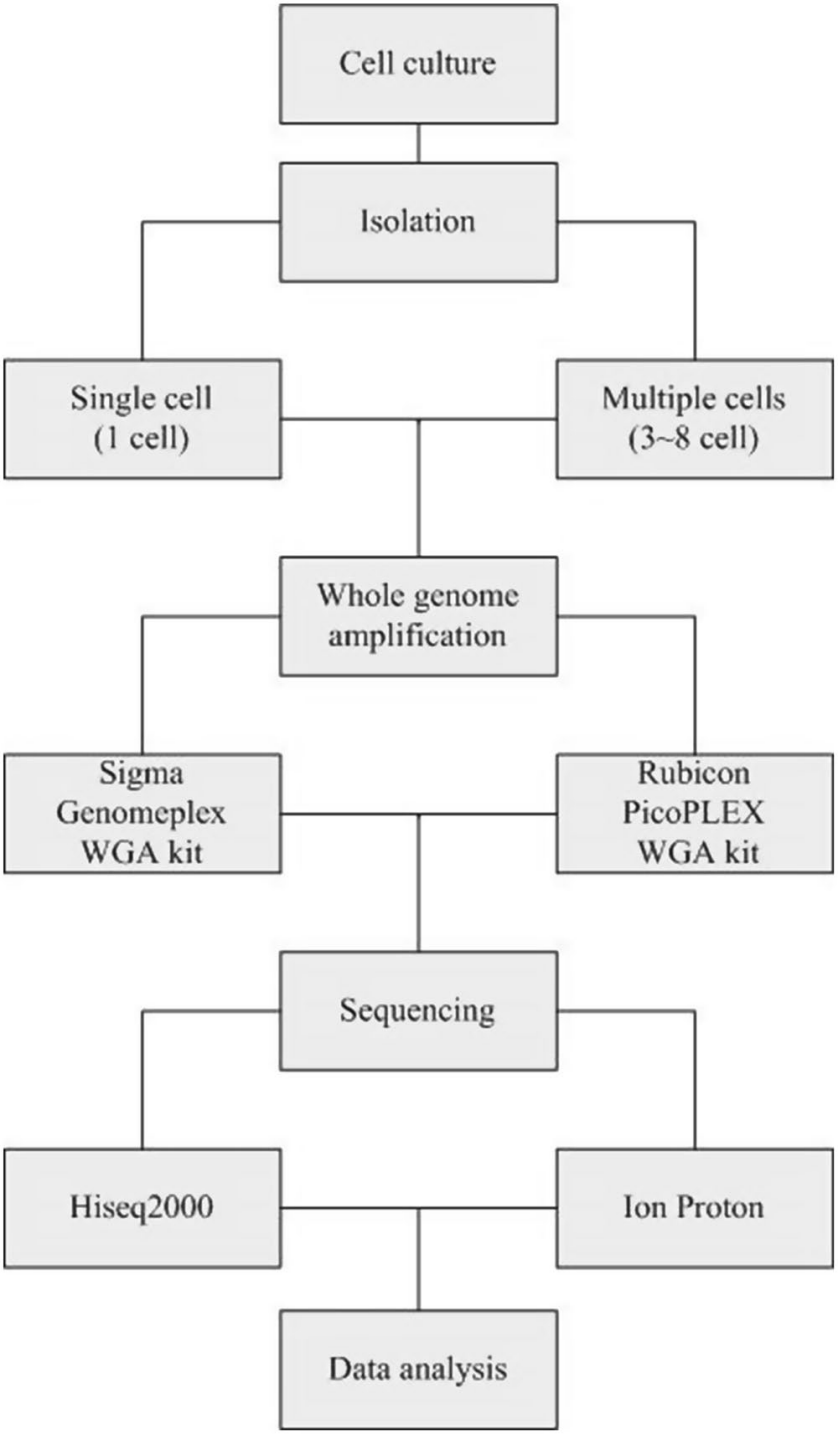
Flow chart. The 11 cell lines were all sequenced in the same sequencing process.

The purpose of this experiment was to evaluate the performance of four combinations on two sequencing platforms. The sample set contained 11 single cells and 11 multiple cells from 11 cell lines (Coriell, New Jersey, US). A total of 88 WGA reactions were conducted based on 3 different experimental factors as follow: cell number (single cell or multiple cells), WGA kit (WGA4 kit or PicoPLEX WGA Kit) and sequencing platform (Ion Proton or HiSeq2000). The flow chart shows the procedure in sample preparation, WGA, sequencing and data analysis (Fig. 1). Sequencing libraries were constructed following Ion Proton and HiSeq2000 sequencing library preparation protocols and then sequenced by Ion Proton and HiSeq2000 platforms. The ability of two WGA kits to detect CNVs was assessed. Additionally, the results of CNVs detection were also compared with the confirmed karyotype of Affymetrix Genome-Wide Human SNP Array 6.0 (Thermo Fisher Scientific, Waltham, MA, USA)^9^.

### Cell information and isolation

Eleven B-lymphocyte cell lines (Coriell, New Jersey, US) were collected individually. All cell lines were confirmed by karyotype analysis after cell line submission to CCR (Coriell Cell Repository, New Jersey, US). Their karyotypes include ten CNVs, one aneuploid and one unbalanced translocation (t (1;6) (1qter >1p36::6p23 >6pter; 6qter >6p23::1p36 >1pter)). The unbalanced translocation was regarded as the negative sample. All details are available on the online support web: https://catalog.coriell.org/ (Supplementary data, Table I). Single cells were isolated as described previously^5^. Briefly, a mouth pipette was used under a microscope, a single cell or 3~8 cells were picked up randomly, washed three or more times with D-PBS (Life Technologies, Carlsbad, US), and then transferred into a PCR tube with eluting in new D-PBS, the final volume was approximately 3 μL. After each transfer, the pipette was validated by microscopy to ensure no cell residues remained.

### WGA with two different kits

Two commercial kits were employed, WGA4 kit and PicoPLEX WGA Kit. All experimental operations conformed to the manufacturers’ instructions strictly. D-PBS (3 μl) was used as a negative control in each set of reactions, in addition to the positive control supplied at the first test of kits. The concentration of WGA products was measured using a Qubit dsDNA Broad-Range Assay kit (Life technologies, Carlsbad, USA) with a Qubit 2.0 Fluorometer (Life technologies, Carlsbad, USA). The product size was determined by loading 1 μL of the final reaction product onto a 1.5% agarose gel. According to manufacturers’ instructions, the DNA size should range from 100 to 1,000 bp, with the mean size of approximately 400 bp.

### Library preparation and DNA sequencing

In this study, the HiSeq2000 and Ion Proton sequencer were used as the sequencing platforms. GC-bias can be introduced at several processes of sequencing, e.g., PCR amplification of library, cluster amplification, and the sequencing process^10^. Among these factors, library amplification by PCR has a primary role in generating GC-bias^10^. To avoid this type of bias, we chose a PCR-free strategy that did not require high DNA input and enrichment PCR. Our experimental operations followed the manufacturers’ protocols strictly and only reduced the input of DNA. A TruSeq DNA PCR-Free Library Preparation Kit (Cat. FC-121-3003, Illumina, San Diego, CA, US) and an Ion Xpress Plus Fragment Library Kit (Cat. 4471269, Life technologies, Carlsbad, USA) were applied for PCR-free library preparation. The kit protocols are supplied on their official website, which do not require high DNA input and enrichment PCR. We prepared the libraries starting with 500 ng of total amplified DNA for each sample. First, all samples were diluted in Tris-EDTA buffer (TE-buffer) for a total volume of 80 μl in a 0.2 ml PCR plate with a plastic stick to fragment by an LE220 Focused Ultra sonicator with Adaptive Focused Acoustics (AFA) technology (Covaris, Woburn, US). Second, the end-repaired and 3′-dA addition steps were performed. Third, adaptor ligation was conducted in the Illumina Library preparation. The Life Tech Library only went through the end-repaired process and then was brought to the next step for P1/PN adaptor ligation, with a nick-translation supplied ultimately. Last, we quantified the yield of libraries and pooled samples together to make a final library. Whole-genome sequencing was performed on Hiseq2000 and Ion Proton. Forty-nine base pair single-end reads were generated using a TruSeq SBS Kit v3-HS (Cat. FC-401-3002; Illumina, San Diego, CA, US) in the HiSeq2000 sequencer, and up to 200 bp single-end reads were generated using an Ion PI Sequencing 200 Kit v3 (Cat. 4488315; Life technologies, Carlsbad, USA) in the Ion Proton platform.

### Alignment

On the HiSeq2000 platform, low quality reads (Phred quality score <20) and the first 20 bp of each read were trimmed. Subsequently, the reads were aligned to the reference genome (GRCh37, UCSC release hg19) using the Burrow-Wheeler-Aligner (BWA) v0.7.7a (bwa aln -l 15 -t 12) algorithm^11^. The alignment result from the SAM file (v1.4-r985) included the mapping read information and the unique non-duplication reads relying on the FLAG (combination of bitwise FLAGs), POS (1-based leftmost mapping position of the first matching base) and MAPQ (mapping quality) values.

On the Ion Proton platform, because the Ion Torrent sequencer only produces single-end (SE) reads that vary in length, we choose the reasonable read length of more than 30 bp for the following analysis. After trimming and filtering processes, Torrent Suite Software (TSS) v 3.4.1 (http://github.com/iontorrent/tmap) was employed to perform the alignment and resulted in bam format. The mapping parameter (tmap mapall -v -Y -u -o 2 -a 0 -n 6 stage1 map4) and the alignment output model parameter in the mapping methods were set as “map all” and “0”, respectively. “map all” indicates multi-mapping procedure, whereas “0” indicates output the unique best hit reads. After removing the duplication on POS, the unique non-duplication reads were used for further analysis.

### GC-bias calculation

GC content bias is the proportion of G and C bases in a specific region compared with that reported previously^12^, which describes the bias resulting from the GC content. The bias leads to abnormal sequencing depth in a specific genomic region, which potentially influences the uniformity of read distribution. Moreover, two primary categories are based on NGS for CNVs-detection methods: the pair-end mapping (PEM) and the depth of coverage (DOC)^13,14^. Most CNVs detection tools are universally designed based on the DOC methods^14^. Coverage of depth depending on the GC content can complicate the accuracy of CNVs detection. To describe the GC-bias in WGA, we referred to the method in the article of Nora Rieber^15^.

Let *R*_1_*, R*_2_*… R*_*w*_ represent the unique non-duplication mapped reads that align to the *W* windows.

1 $${\rm{Total}}\,{\rm{variance}}:\,\mathrm{TV}=\frac{\,1\,}{\,W\,}{\sum }_{w}{({R}_{W}-M)}^{2}$$

2 $${\rm{Variance}}\,{\rm{after}}\,{\rm{G}}+{\rm{C}}\,{\rm{loess}}\,{\rm{fit}}:\,LV=\frac{\,1\,}{\,W\,}{\sum }_{w}{({R}_{W}-{L}_{W})}^{2}$$

3 $${\rm{Contribution}}\,{\rm{of}}\,{\rm{G}}+{\rm{C}}\,{\rm{bias}}\,{\rm{to}}\,{\rm{total}}\,{\rm{variance}}:{\rm{\Delta }}{R}_{GC}=1-\frac{LV}{TV}$$

where *M* represents the average number of unique non-duplication mapped reads on each autosome window, *L*_*w*_ is obtained via a loess local regression fit of the unique non-duplication mapped reads against the G + C content, and Δ*R*_*GC*_ is the quantitative value of GC-bias. Small values of Δ*R*_*GC*_ indicate the GC-bias is less serious. However, Δ*R*_*GC*_ is a relative measure and can be influenced by WGA uniformity.

### Data analyses

The windows selection was performed referring to previous reports, GC-bias correction and copy number analysis^12^. In brief, the reference genome (GRCh37, UCSC release hg19) was divided into sliding SE50 simulated reads and mapped back to the origin reference genome with a maximum of two mismatches. Among the 100 K simulated unique mapped reads in continuous windows, we allowed 20 K overlapping reads to exist. The GC content of each window was calculated and used for the GC-bias correction. The normalized depth ratio (NDR) is the unique mapped non-duplication reads of each window divided by the total average unique mapped non-duplication reads, which was used to calculate the coverage and evaluate the reproducibility and uniformity. Additionally, we referred to the algorithm from Zhang *et al*.^12^ to detect CNVs. To remain as close to the characteristics of the human reference genome as possible, we used the optimized dynamic window size to call CNVs. After the GC-bias correction and binary segmentation, we discerned the CNVs breakpoints. Sensitivity and specificity were calculated as follow:

4 $${Sensitivity}=\frac{{TPR}}{({TPR}+{FNR})}$$

5 $${Specificity}=\frac{{TNR}}{({TNR}+{FPR})}$$

where FNR is short for false negative rate which equal to the false negative signal number divided by the total true positive signal number. FPR is short for false positive rate which equal to the signal number divided by the total true positive signal number. TNR is short for negative true negative rate which equal to the true negative signal number divided by the total true negative signal number. TPR is short for true positive rate which equal to the true positive signal number divided by the total true positive signal number. The difference in different groups was analysed by one-way ANOVA^16^. We also performed the Mann–Whitney-Wilcoxon test to assess the variation between two groups. Differences yielding *P*-values below or equal to 0.05 were considered significant. Numbers given before the ‘±’ symbol in results indicate the average value, and numbers given after the ‘±’ symbol indicate standard deviation.

### Ethical approval

This article does not contain any studies with human participants or animals performed by any of the authors.

## Results

### Comparison of amplification time and yield

The amplification yield was compared using the two WGA kits in the final volume of 75 μL of amplification product. The WGA4 kit had the WGA product at the concentration of 72.98 ± 17.81 ng/μL, whereas the PicoPLEX WGA Kit had the WGA product at the concentration of 37.56 ± 4.96 ng/μL. The yield of different cell numbers using the same WGA kit was not different, but a significant difference was detected between the two WGA kits. Additionally, approximately 4.5 h with the WGA4 kit and 2.5 h with the PicoPLEX WGA Kit were required to finish the WGA procedure. Comparatively, less time was consumed with the PicoPLEX WGA Kit to obtain sufficient yield for library construction.

### Data production

To reduce the effect of sequencing depth on the comparison of each combination, we randomly extracted 2 million clean reads from the total data of each sample (Supplementary Table II, HiSeq2000, Supplementary Table III, Proton). The extraction strategy and reason are described previously^7^. Table 1 shows the mean basic statistics of both platforms. We found that the mean unique mapping rate (58.72%) of PicoPLEX WGA Kit was lower than that of WGA4 kit (62.43%) on the HiSeq2000 platform (Supplementary Fig. S1). On the Proton platform, the average unique mapping rate of WGA4 kit was 91.23% and that of the PicoPLEX WGA Kit was 91.36% (Supplementary Fig. S1), the mapping rate of WGA4 kit was much higher than PicoPLEX WGA Kit on the Hiseq2000 platform.

**Table 1:** Global Average Statistics of Sequencing and Mapping of different Platforms and Kits.

Platform	Kit	Cell Number	Raw Reads (M)	Sample Number	Unique Mapping Rate (%)	Coverage (%)	GC Content (%)	Duplication Reads Rate (%)	Mismatch Rate (%)	Deletion Rate (%)	Insertion Rate (%)	Error Rate (%)
HiSeq 2000	WGA4 kit	1	2	11	63.01	0.30	39.67	5.34	1.76	0.03	0.02	1.81
	WGA4 kit	3–8	2	11	61.85	0.31	39.74	2.06	1.90	0.03	0.03	1.96
	PicoPLEX WGA Kit	1	2	11	58.47	0.32	44.11	1.18	1.44	0.05	0.06	1.55
	PicoPLEX WGA Kit	3–8	2	11	58.95	0.33	44.08	1.04	1.43	0.04	0.05	1.52
Proton	WGA4 kit	1	2	11	91.31	4.60	42.05	13.43	1.31	0.31	0.46	2.08
	WGA4 kit	3–8	2	11	91.18	5.15	41.68	11.97	1.26	0.30	0.42	1.98
	PicoPLEX WGA Kit	1	2	11	90.96	5.24	45.29	10.77	1.47	0.35	0.43	2.25
	PicoPLEX WGA Kit	3–8	2	11	91.83	5.47	45.15	10.26	1.49	0.37	0.43	2.29

To gain further insights into the data quality, we investigated the discordantly mapped reads derived from different libraries and sequencing processes. The mismatch rate, deletion rate and insertion rate are a series of important parameters to consider for calling single-nucleotide variants (SNVs). Based on the alignment results and the Compact Idiosyncratic Gapped Alignment Report (CIGAR), we encoded matches and mismatches with an ‘M’, insertions with an ‘I’ and deletions with a ‘D’. Subsequently, we defined ErrorRate as the sum of mismatch rate, deletion rate and insertion rate (Table 1). The results of variance analysis (Supplementary Fig. S2) suggested that the PicoPLEX WGA Kit had a lower ErrorRate (*P* < 0.01) than that of the WGA4 kit on the HiSeq2000 platform independent of cell number. The results were reversed on the Ion Proton platform. Furthermore, the ErrorRate of Hiseq2000 was lower than that of Ion Proton with the same WGA kit.

However, whether the map rate of Ion Proton was higher than that of Hiseq2000 or the difference between the mismatch rate, insertion rate and deletion rate was significant could not be determined because the two sequencing platforms were not comparable because of the different alignment methods used and different sequencing principles^17^.

### GC-bias of four combinations

**Figure 2:**
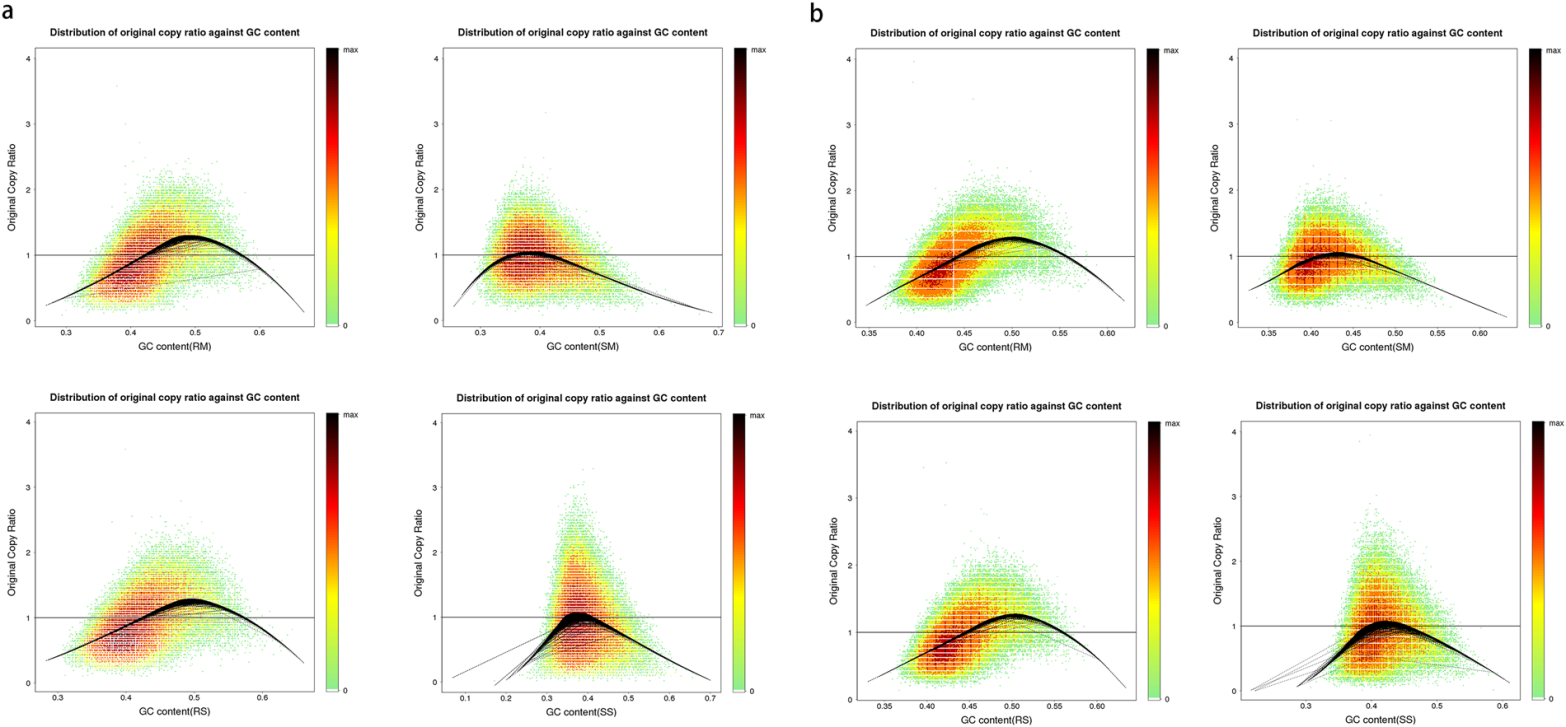
GC plots for HiSeq2000 (**a**) and Proton (**b**) platforms. A heat map describes rates for each (GC, Original copy ratio) pair. Smoothed loess curves (black line) are fitted to represent the local original copy ratio trend. RS, RM, SS, SM are four combinations. RS is short for Rubicon PicoPLEX WGA Kit and single cell, RM is short for Rubicon PicoPLEX WGA Kit and multiple cells, SS is short for Sigma-Aldrich WGA4 kit and single cell, SM is short for Sigma-Aldrich WGA4 kit and multiple cells.

Generally, GC-bias is considered an important factor that complicates data analysis. The plot of the NDR at various genomic regions versus the GC content showed that the average GC content was 39.70% on HiSeq2000 and 41.86% on Ion Proton using the WGA4 kit, which were values very close to those of the reference genome (41.9%). By contrast, the average GC content was 44.10% on HiSeq2000 and 45.22% on Ion Proton with the PicoPLEX WGA Kit (Fig. 2). These results demonstrated the amplification preference of the PicoPLEX WGA Kit on GC-rich regions.

**Figure 3:**
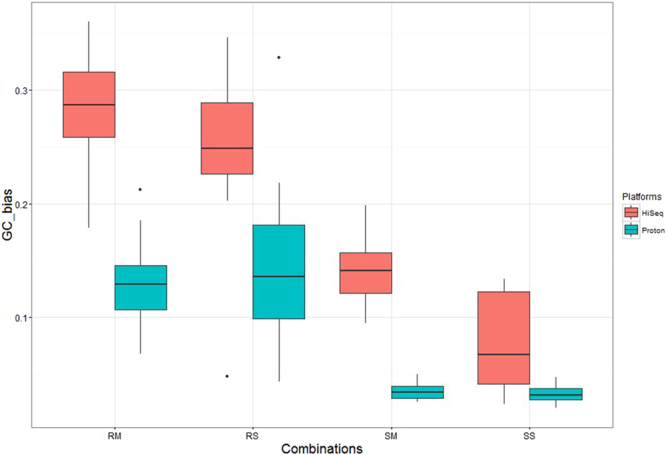
Values of ΔR_GC_ for the four combinations between Hiseq2000 and Proton platforms. The box-plot represents the correlation of 11 cell lines used in this study for HiSeq2000 and Proton platforms. RS, RM, SS, SM are four combinations. RS is short for Rubicon PicoPLEX WGA Kit and single cell, RM is short for Rubicon PicoPLEX WGA Kit and multiple cells, SS is short for Sigma-Aldrich WGA4 kit and single cell, SM is short for Sigma-Aldrich WGA4 kit and multiple cells.

Commonly, Δ*R*_GC_ is used to quantify GC-bias, and a small ΔR_GC_ value indicates reduced GC-bias. We analysed the ΔR_GC_ value for the four combinations on the two platforms (Fig. 3). On the HiSeq2000 platform, the values of ΔR_GC_ from PicoPLEX WGA Kit amplified data were 0.25 ± 0.08 and 0.29 ± 0.05 for single cell and multiple cells, respectively, whereas the values were 0.08 ± 0.04 and 0.14 ± 0.03 for single cell and multiple cells, respectively, of WGA4 kit amplified data. Conclusively, SS had significantly less GC-bias than that of RS (*P* < 0.05), and SM had less GC-bias than that of RM (*P* < 0.05). Thus, data generated with the WGA4 kit had less GC-bias than the data generated with the PicoPLEX WGA Kit on the Hiseq2000 platform. On the Ion Proton platform, the values of ΔR_GC_ from PicoPLEX® amplified data were 0.13 ± 0.04 for RM and 0.15 ± 0.08 for RS. The values of ΔR_GC_ from WGA4 kit amplified data were 0.04 ± 0.01 for SM and 0.03 ± 0.01 for SS. To summarize, data generated with the WGA4 kit had less GC-bias than data generated with the PicoPLEX WGA Kit for single cell (*P* < 0.05) and multiple cells (*P* < 0.05).

**Figure 4:**
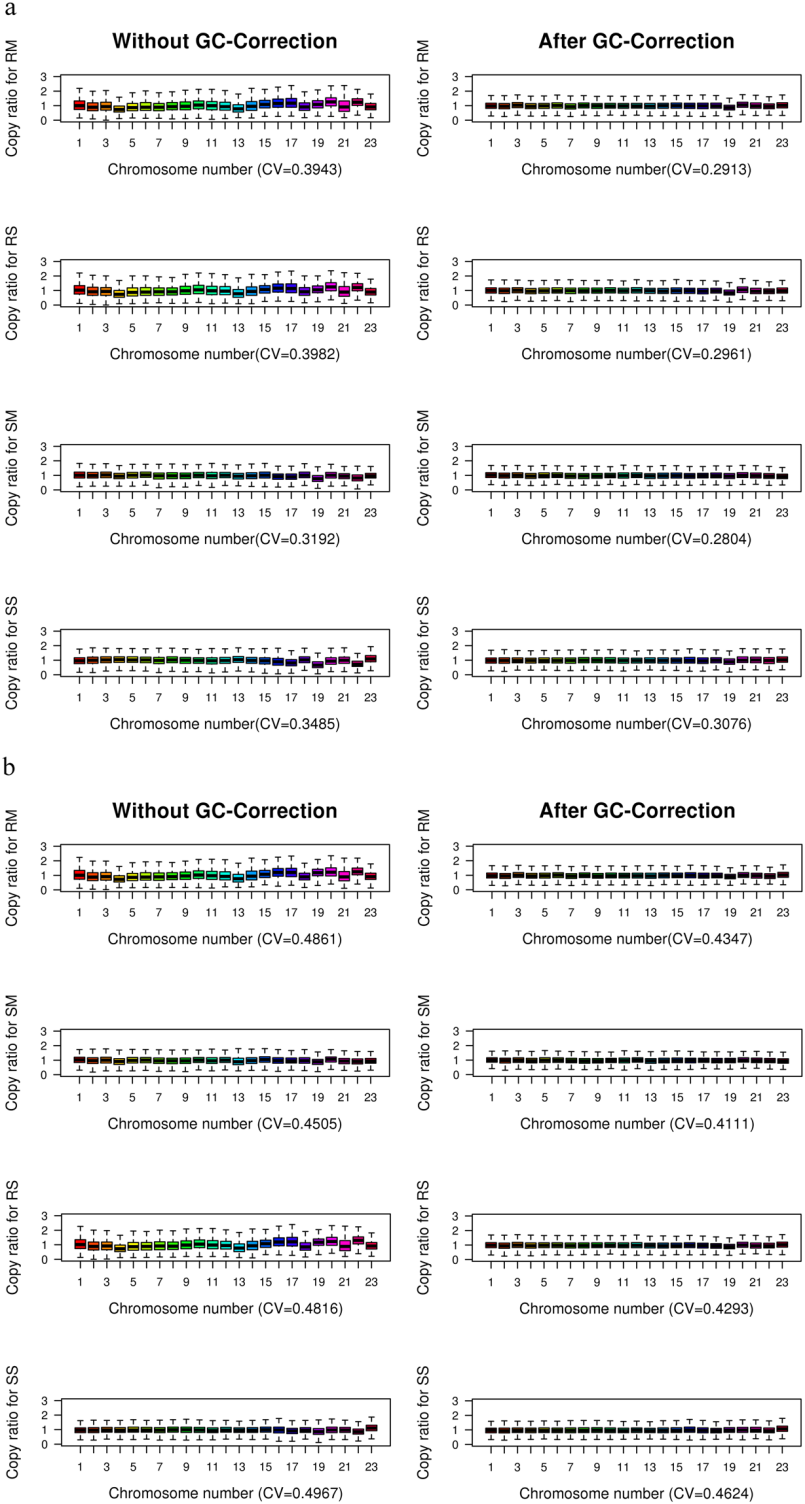
Distribution of NDR values for the four combinations across the whole genome on HiSeq2000 (**a**) and Proton (**b**) platforms. Box plot represents NDR values in 124,011 windows for the same sample. x-axis is Chromosome number; y-axis is NDR values. The left and right represent the comparison without GC-correction and after GC-correction, respectively, for the same combination. The CV is the coefficient of variation of NDR across the whole genome. RS, RM, SS, SM are four combinations. RS is short for Rubicon PicoPLEX WGA Kit and single cell, RM is short for Rubicon PicoPLEX WGA Kit and multiple cells, SS is short for Sigma-Aldrich WGA4 kit and single cell, SM is short for Sigma-Aldrich WGA4 kit and multiple cells.

Based on this discovery, a weighted correction strategy could be used to remove the GC-bias (Fig. 4), which was reported to correct more than 99.9% of the GC-bias^12^.

### Reproducibility Evaluation

Reproducibility is the ability to reproduce experimental results, either by the sample type or experimental combination, and is particularly important when the amount of DNA is typically at a picogram level. In this study, we used Pearson’s correlation coefficient of the NDR on a selected window along the autosome to quantify the reproducibility between two representative combinations. The correlation value matrix was calculated between any two cell lines among the 11 cell lines.

On the HiSeq2000 platform, the correlation values of PicoPLEX WGA Kit amplification data were 0.62 ± 0.18 and 0.79 ± 0.03 for single cell and multiple cells, respectively; whereas the values were 0.28 ± 0.08 and 0.57 ± 0.06 for single cell and multiple cells, respectively, when using the WGA4 kit. RS had significantly better reproducibility than that of SS (*P* < 0.05), and RM also had better reproducibility than that of SM (*P* < 0.05).

**Figure 5:**
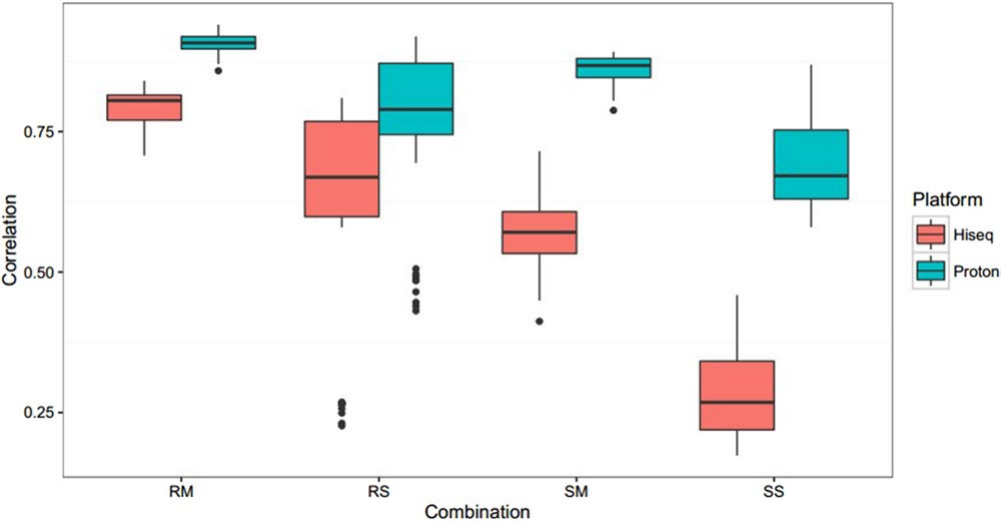
Reproducibility of the four combinations between HiSeq2000 and Proton platforms. The box-plot represents the correlation of 11 cell lines used in this study for HiSeq2000 and Proton platforms. RS, RM, SS, SM are four combinations. RS is short for Rubicon PicoPLEX WGA Kit and single cell, RM is short for Rubicon PicoPLEX WGA Kit and multiple cells, SS is short for Sigma-Aldrich WGA4 kit and single cell, SM is short for Sigma-Aldrich WGA4 kit and multiple cells.

On the Proton platform, the correlation values of PicoPLEX WGA Kit amplification data were 0.76 ± 0.15 and 0.91 ± 0.02 for single cell and multiple cells, respectively; whereas the values were 0.69 ± 0.08 and 0.86 ± 0.03 for single cell and multiple cells, respectively, when using the WGA4 kit (Fig. 5). RS had significantly better reproducibility than that of SS (*P* < 0.05), and RM had significantly better reproducibility than that of SM (*P* < 0.05). These results demonstrated that the PicoPLEX WGA Kit outperformed WGA4 kit on reproducibility for the corresponding cell number on both Hiseq2000 and Ion Proton platforms.

### Genome coverage uniformity

**Figure 6:**
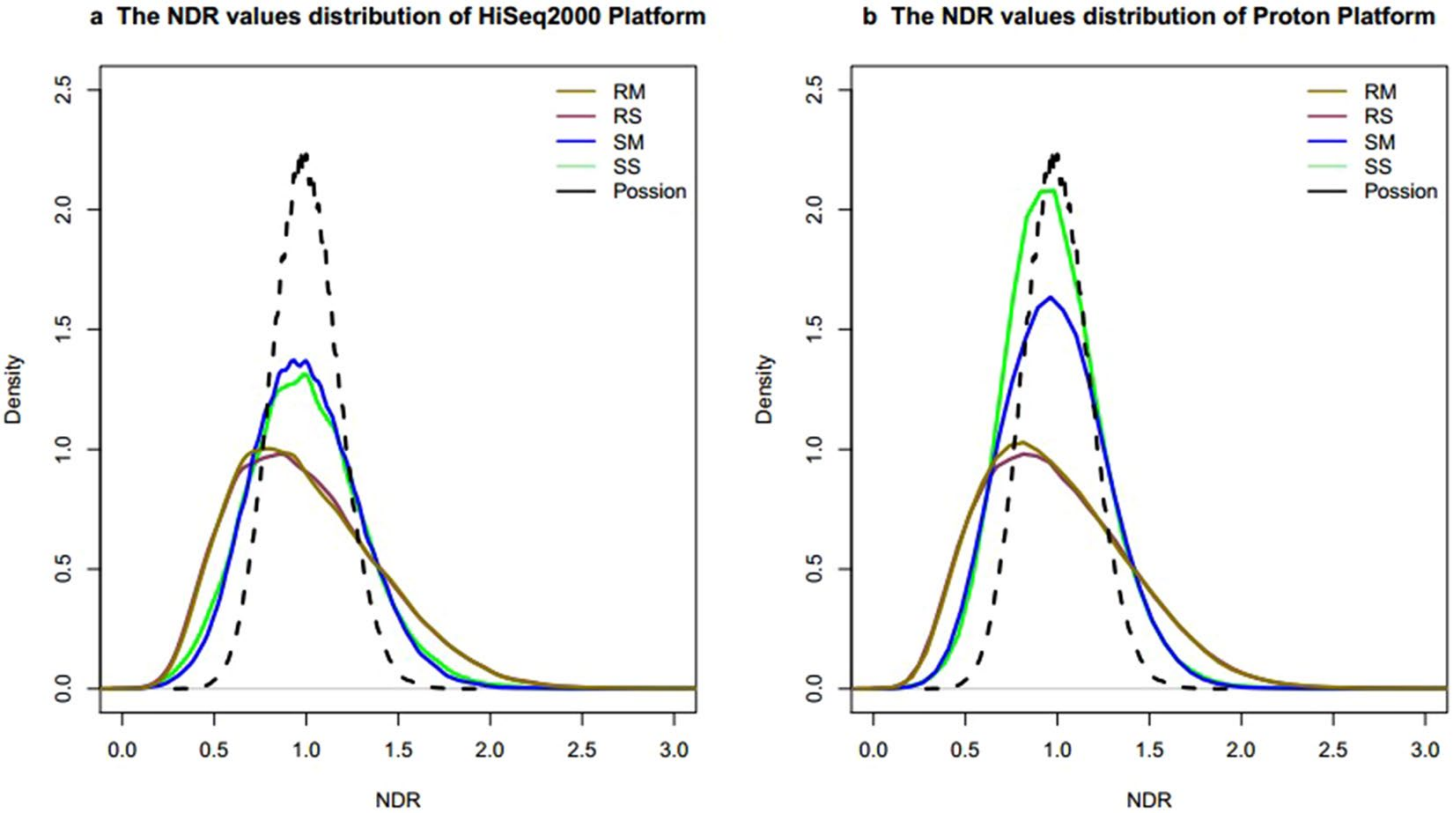
Overview of the NDR value distribution for the four combinations. The NDR value is defined as the number of reads in each window to the mean number of reads in autosomal windows. The dashed curve is plotted using the simulated data (124,011 dots), which conformed to the Poisson distribution (λ = 30) and was normalized by dividing by 30. RS, RM, SS, SM are four combinations. RS is short for Rubicon PicoPLEX WGA Kit and single cell, RM is short for Rubicon PicoPLEX WGA Kit and multiple cells, SS is short for Sigma-Aldrich WGA4 kit and single cell, SM is short for Sigma-Aldrich WGA4 kit and multiple cells.

Coverage depth has been widely employed in different CNVs calling algorithms, and uniformity of WGA product is important to coverage depth and CNVs detection. Therefore, we characterized the uniformity by comparing the uniformity of reads distribution using the extracted data mentioned above. We simulated the theoretical sequencing depth distribution, which followed the Poisson distribution (124,011 dots, λ = 30), and normalized it by dividing by 30. Previously, we found that the distribution of data from the WGA4 kit was close to the theoretical one on the two sequencing platforms; whereas bias was observed in the data from the PicoPLEX WGAKit (Fig. 6). The CV value effectively described the relative variance of chromosomal depth, uniformity, and overall GC-bias in previous studies^6^. We also used the CV value to quantify the uniformity of NDR and a box-plot to display the whole genome variation (Supplementary Fig. S3). On HiSeq2000, the CV values were 0.31 ± 0.01 in RM, 0.29 ± 0.02 in SM, 0.39 ± 0.14 in RS and 0.40 ± 0.06 in SS. On Proton, the CV values were 0.50 ± 0.04 in RM, 0.45 ± 0.03 in SM, 0.55 ± 0.09 in RS and 0.51 ± 0.06 in SS. The WGA4 kit had significantly better uniformity than that of PicoPLEX WGA Kit for multiple cells (HiSeq2000, *P* < 0.05 and Ion Proton, *P* < 0.05) on the two sequencing platforms. By contrast, the two kits were not different for single cell amplification on either sequencing platform (HiSeq2000, *P* = 0.32 and Ion Proton, *P* = 0.24).

Further, we calculated the linear regression of the four combinations between the two platforms and returned the coefficient of determination. The R^2^ value was 0.61 for RM, 0.78 for RS, 0.40 for SS, and 0.22 for SM. The results for R^2^ values showed that the PicoPLEX WGA Kit had better consistency than that of the WGA4 kit between the two sequencing platforms. In conclusion, the WGA4 kit had better uniformity than that of PicoPLEX WGA Kit on both Hiseq2000 and Ion Proton sequencing platforms. With expectations, the CV value of multiple cells was lower than that of single cell independent of WGA kit and sequencing platforms.

### Copy number variations analysis

**Figure 7:**
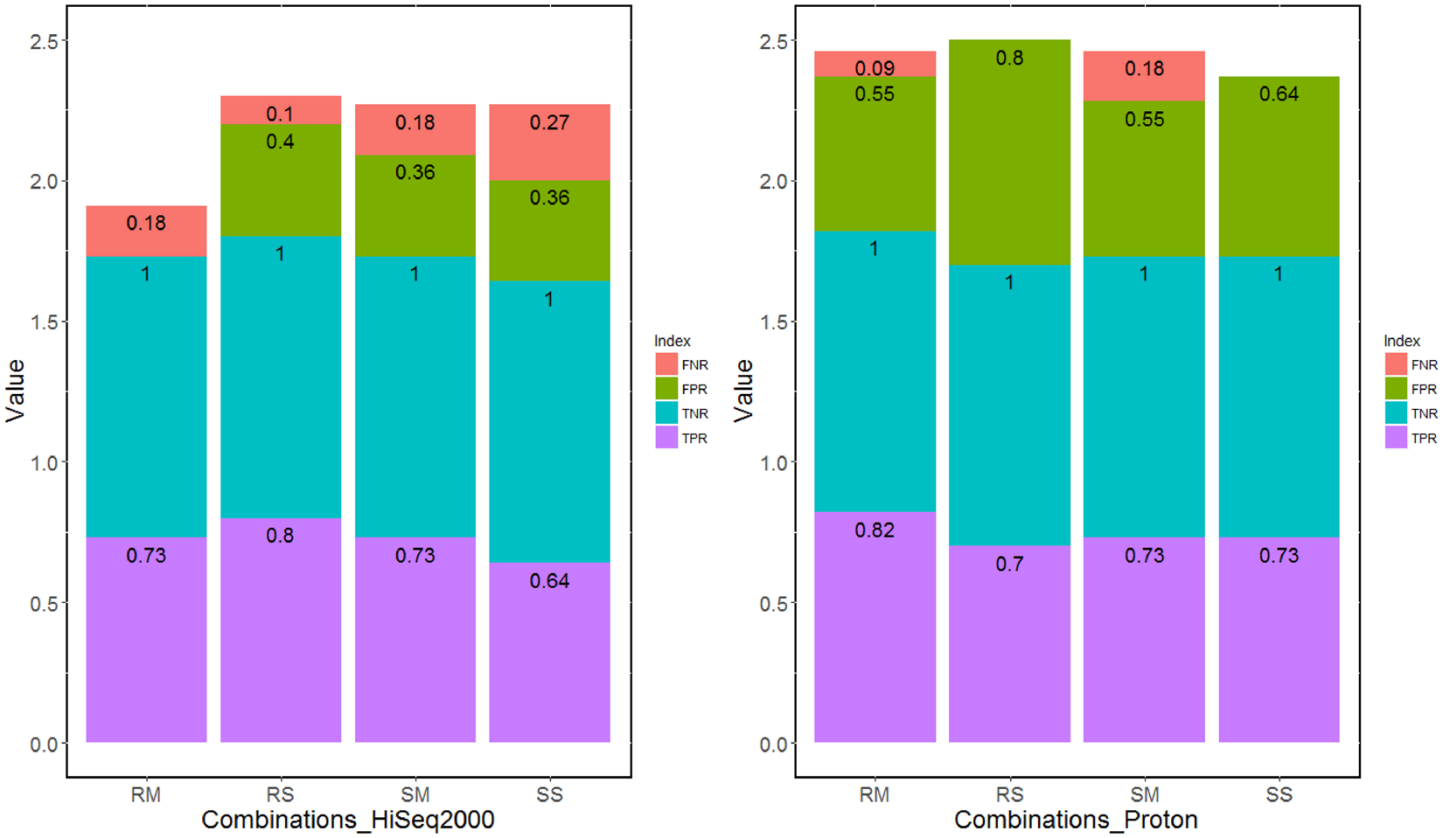
CNV detection on HiSeq2000 (**a**) and Proton (**b**) platforms. FNR is short for false negative rate which equal to the false negative signal number divided by the total true positive signal number. FPR is short for false positive rate which equal to the signal number divided by the total true positive signal number. TNR is short for negative true negative rate which equal to the true negative signal number divided by the total true negative signal number. TPR is short for true positive rate which equal to the true positive signal number divided by the total true positive signal number. RS, RM, SS, SM are four combinations. RS is short for Rubicon PicoPLEX WGA Kit and single cell, RM is short for Rubicon PicoPLEX WGA Kit and multiple cells, SS is short for Sigma-Aldrich WGA4 kit and single cell, SM is short for Sigma-Aldrich WGA4 kit and multiple cells.

Compared with the results of the SNP array, we calculated the sensitivity and specificity of the four combinations on the two sequencing platforms (Fig. 7). The RS combination of CL01 was excluded on the two platforms because it showed a significantly erratic fluctuation, which did not meet the requirements of CNV detection. Conclusively, on the Hiseq2000 platform, the average sensitivity was 0.85 (0.80 for RM and 0.89 for RS) and the average specificity was 0.86 (1.00 for RM and 0.71 for RS) using the PicoPLEX WGA Kit, whereas the average sensitivity was 0.75 (0.80 for SM and 0.70 for SS) and the average specificity was 0.74 (0.74 for SM and 0.74 for SS) using the WGA4 kit. This result suggested that the PicoPLEX WGA Kit performed slightly superior the WGA4 kit on sensitivity on the HiSeq2000 platform. However, on specificity, the PicoPLEX WGA Kit performed slightly superior the WGA4 kit only when using multiple cells. When using single cell, by contrast, the performance of the WGA4 kit was slightly superior to that of the PicoPLEX WGA Kit. Additionally, on the Ion Proton platform, the average sensitivity was 0.84 (0.90 for RM and 0.78 for RS) and the average specificity was 0.60 (0.65 for RM and 0.56 for RS) using the PicoPLEX WGA Kit, whereas the average sensitivity was 0.80 (0.80 for SM and 0.80 for SS) and the average specificity was 0.63 (0.65 for SM and 0.61 for SS) using the WGA4 kit. Based on this result, PicoPLEX WGA Kit performed slightly superior the WGA4 kit on sensitivity, and these two kits had the same specificity when using multiple cells. However, when using single cell, by contrast, the WGA4 kit performed superior to the PicoPLEX WGA Kit on both sensitivity and specificity. Generally, on these two sequencing platforms, depending on cell number, the performance of the two WGA kits was different for both sensitivity and specificity. All results of the CNVs detection are listed in Supplementary Tables IV and V.

## Discussion

Our studies presented a comprehensive comparison for four combinations based on two sequencing platforms using the same sample set. Considering higher reproducibility, lower sequencing error, better uniformity, and comparable sensitivity and specificity, the PicoPLEX WGA Kit was the best choice for multiple cells WGA on the HiSeq2000 sequencing platform. However, on the Proton platform, the WGA4 kit showed better uniformity, lower sequencing error, and higher uniformity but lower reproducibility than the PicoPLEX WGA Kit^18^. Additionally, the PicoPLEX WGA Kit and the WGA4 kit were both highly reproducible, which indicated the two kits could be used to study cell-to-cell genomics on the Ion Proton platform.

Our results showed that the expected variations could be identified without control samples, although a few false positive signals were also called. Those false positive signals were likely caused by artificial biases induced by uneven amplification of genomic regions^19^, particularly on the sex chromosome. When researchers want to develop new bioinformatics tools, they can systematically summarize the patterns of bias and reduce those false signals by building a filtering set. Further, researchers also can reduce the noise level of data by filtering specific regions leading to the sequencing bias, such as satellites and centromeric and telomeric repeats^20^.

Previous study revealed that either the WGA step or the sequencing step might lead to GC content bias in the single-cell whole genome sequencing process^21^. Different sequencing platforms also show different levels of GC content bias^22^. In this study, we first quantified the GC-bias in different combinations. We found that the PicoPLEX WGA Kit had higher GC-bias values than those of the WGA4 kit. Using an index, researchers can quantify the degree of GC-bias correction in developing a more robust detection pipeline^21^.

In this study, we did not consider sample processing time, reagents consumption, labour costs or sample size. Those parameters might have an important role in technology selection, particularly in the scenario of clinical use. However, rapid advances in sequencing technology are likely to change those parameters in the future. Researchers within the expanding field of single cell research can obtain various experimental parameters from the cell lines before managing a multitude of clinical samples from large trials. In pre-implantation genetic screening (PGS) research, those advantages become more obvious because PGS involves a screening process before implantation for one or more nuclei from oocytes [a polar body or bodies (PBs)] or embryos (blastomere or trophectoderm cells) to detect the chromosomal CNVs^23^, and therefore, SLWGS for identifying CNVs has become common practice in PGS^24^.

## Electronic supplementary material

Supplementary Information
