# Supplementary material for: Comparison of single cell sequencing data between two whole genome amplification methods on two sequencing platforms
Source: Sci Rep. 2018 Mar 21;8:4963. doi: 10.1038/s41598-018-23325-2 (PMC5862989; doi:10.1038/s41598-018-23325-2)
Supplement: Supplementary file 1 — Supplementary Information [file 41598_2018_23325_MOESM1_ESM.pdf]

Comparison of single cell sequencing data between two whole genome amplification methods on two sequencing platforms

DaYang Chen[1,4†], HeFu Zhen[3,4†], Yong Qiu[4†], Ping Liu[4], Peng Zeng[4], Jun Xia[4], QianYu Shi[4], Lin Xie[4], Zhu Zhu[4], Ya Gao[4], GuoDong Huang[4], Jian Wang[4,5], HuanMing Yang[4,5], Fang Chen[2,4]*

1, BGI Education Center, University of Chinese Academy of Sciences, Shenzhen, 518083, China

2, Laboratory of Genomics and Molecular Biomedicine, Department of Biology, University of Copenhagen, DK-2100, Copenhagen, Denmark

3, School of Bioscience and Bioengineering, South China University of Technology, Guangzhou 510006, China.

4, BGI-Shenzhen, Shenzhen, China

5, James D. Watson Institute of Genome Sciences, Hangzhou 310058, China

*Correspondence address. E-mail: Fangchen@genomics.cn

†The authors consider that the first three authors should be regarded as joint First Authors

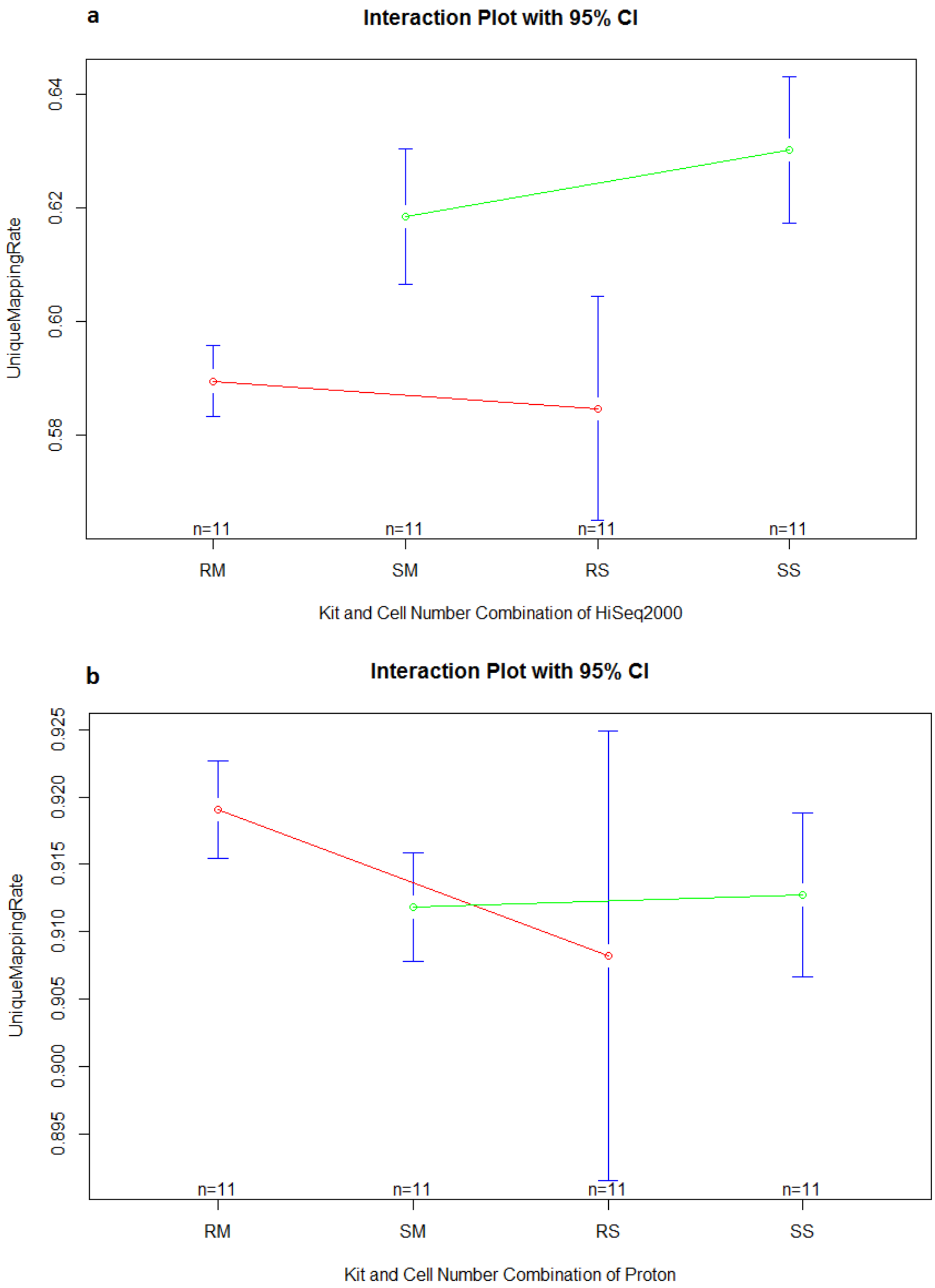

Figure S1 The interactions effect of WGA Kit and Cell Number for HiSeq2000 (a) and Proton (b). The y-axis is the UniqueMappingRate with different Combination. The figure shows average value, sample number and error bar (95% confidence interval). The color-code lines represented data generated on Rubicon PicoPLEX WGA Kit (red) and Sigma-Aldrich WGA4 kit (green) respectively.

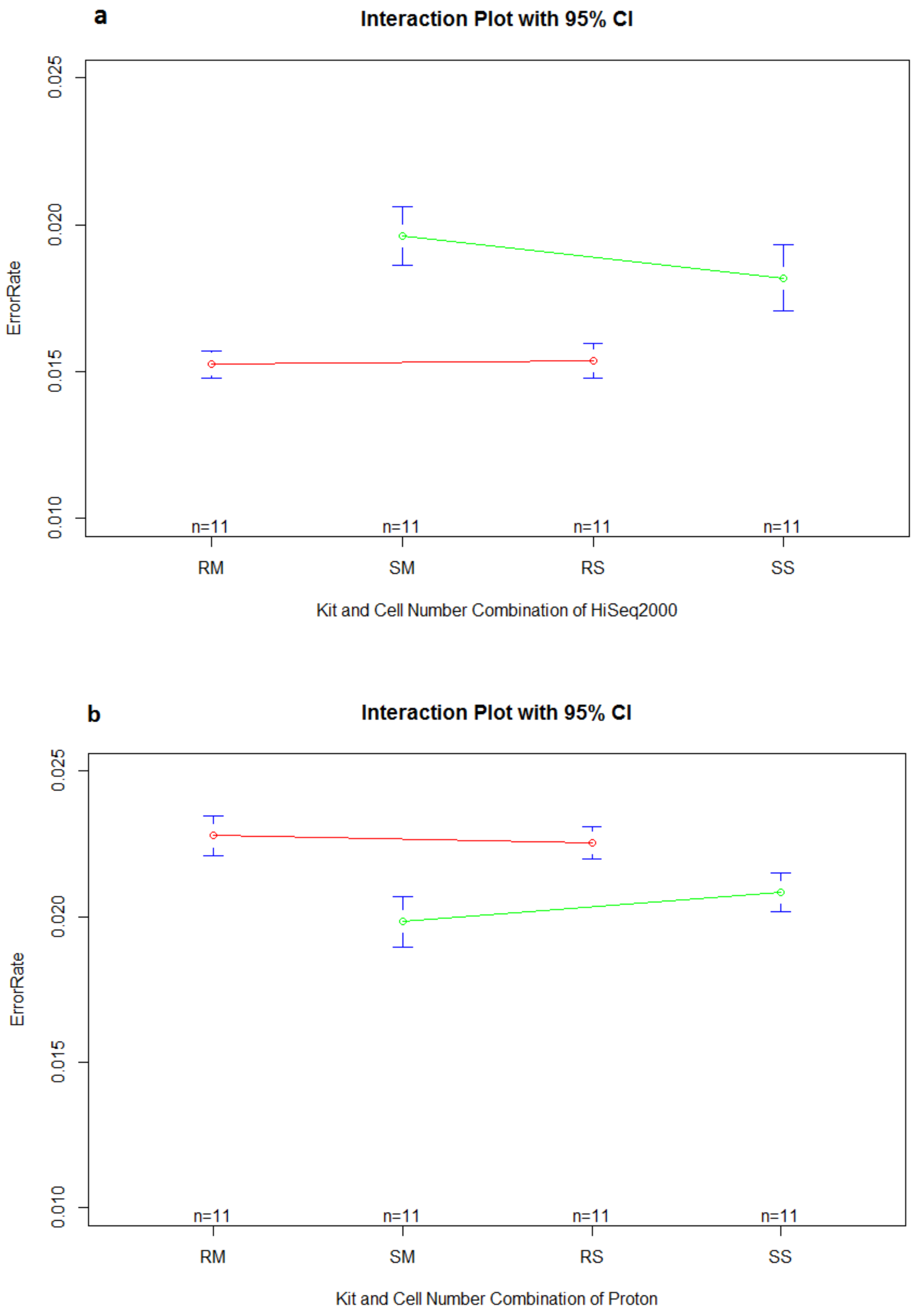

Figure S2 The interaction effect of Kit and Cell Number for HiSeq2000 (a) and Proton (b). The y-axis is the ErrorRate with different combination. The figure shows average value, sample number and error bar (95% confidence interval). The color-code lines represented data generated on Rubicon PicoPLEX WGA Kit (red) and Sigma-Aldrich WGA4 kit (green) respectively.

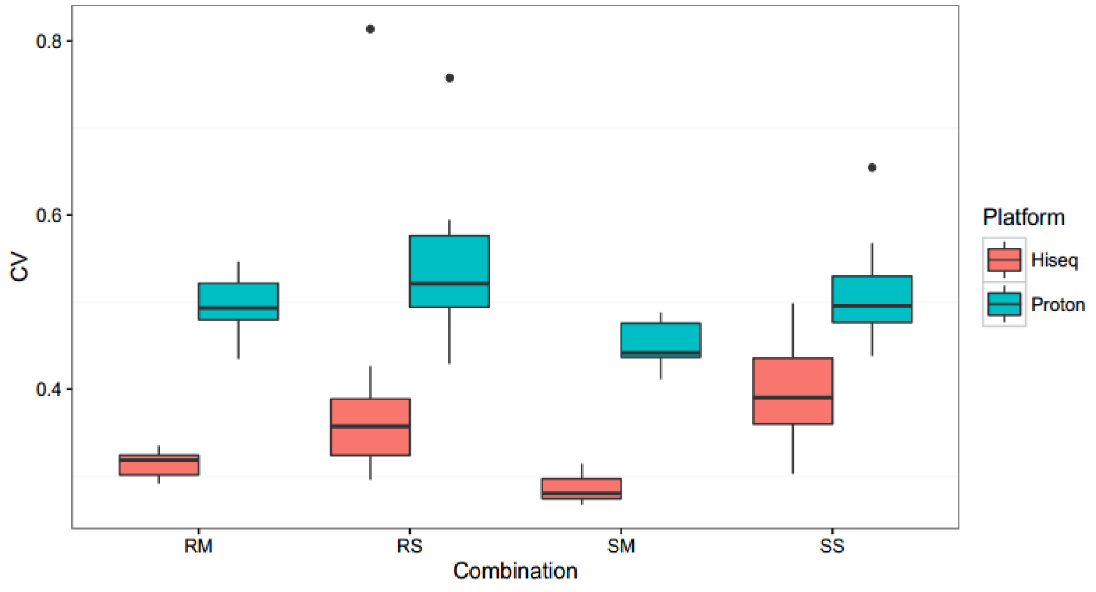

Figure S3 CV value of 11 samples distribution of four combinations for two platforms. The y-axis is the CV value and the x-axis is the different combination. HiSeq2000: RM (0.31 ± 0.01), SM (0.29 ± 0.02), RS (0.39 ± 0.14), SS (0.40 ± 0.06); Ion Proton, RM (0.50 ± 0.04), RS (0.55 ± 0.09), SM (0.45 ± 0.03), SS (0.51 ± 0.06).

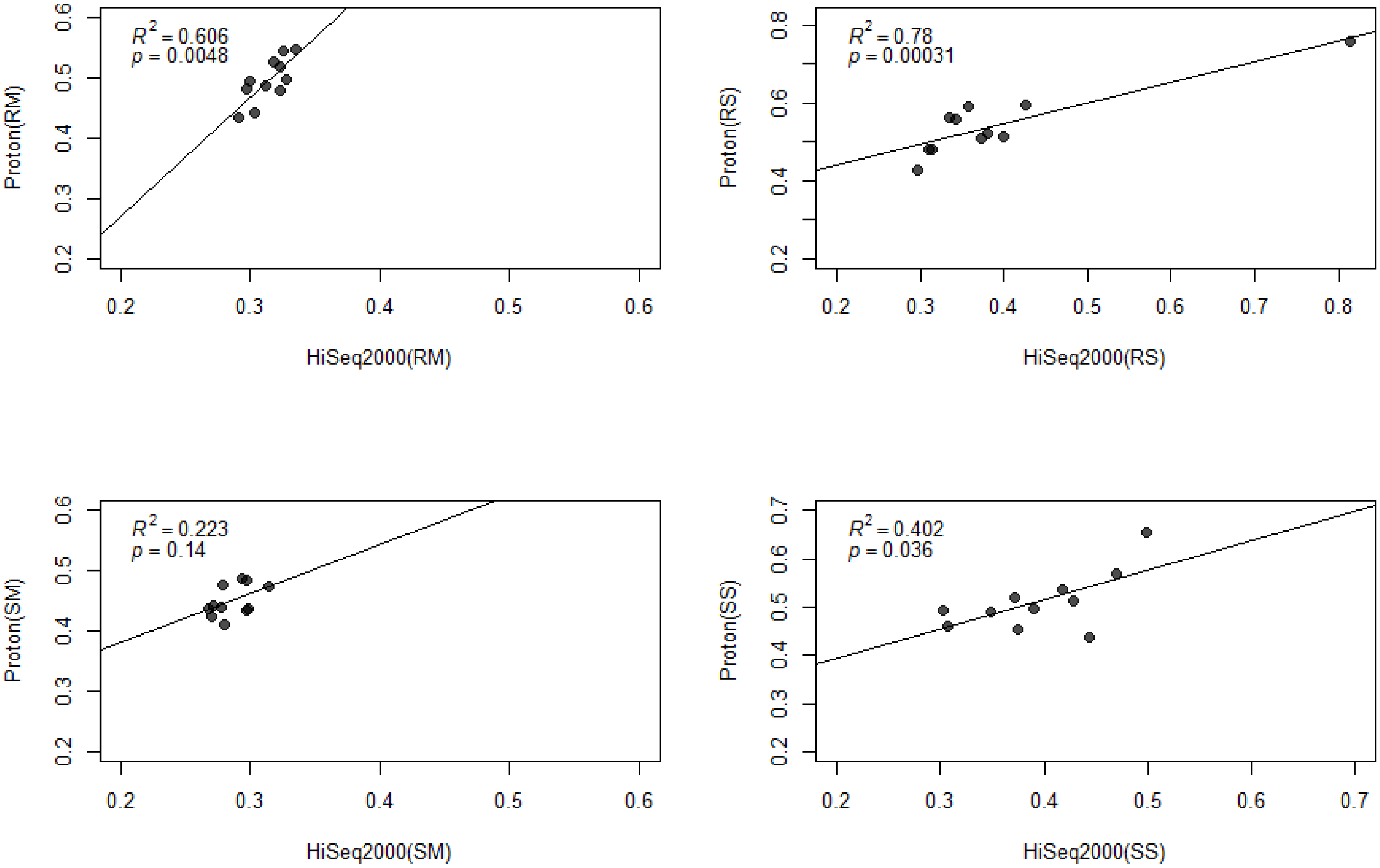

Figure S4 R^2 value of the same combination on two sequencing platforms. The y-axis is the Ion Proton platform and the x-axis is the Hiseq2000 platform. The R^2 value of RM is 0.61, the R^2 value of RS is 0.78, the R^2 value of SS: 0.40, the R^2 value of SM is 0.22.

Table I Information of 11 cell lines from Coriell Institute

Paper ID	CLID	Chromosome	Cell Type	Description	ISCN
CL01	GM13277	chrX	B-Lymphocyte	CHROMOSOME DELETION	46,X,del(X)(q13)
CL02	GM13031	chr17	B-Lymphocyte	CHROMOSOME DELETION	46,XY,del(17)(q21.3q23)[47]/46,XY[3]
CL03	GM11419	chrY	B-Lymphocyte	ANEUPLOID CHROMOSOME NUMBER - NON-TRISOMIC	49,XYYYY
CL04	GM03423	N/A	B-Lymphocyte	TRANSLOCATED CHROMOSOME	46,XX,t(1;6)(1qter>1p36::6p23>6pter; 6qter>6p23::1p36>1pter)
CL05	GM10918	chr2	B-Lymphocyte	CHROMOSOME DELETION	46,XX,del(2)(q34q36)
CL06	GM12662	chrX	B-Lymphocyte	CHROMOSOME DELETION	46,dup(X)(q28),del(Y)(q11.2).ish del(Y)(q11.2)(DXYS129/DXYS153+,SRY+,DYZ3+,DYZ1+,Z43206+)
CL07	GM22364	chr15	B-Lymphocyte	DUPLICATED CHROMOSOME	46,XY,dup(15)(q11q12).ish dup(15)(q11q12)(D15Z1+,GABRB3 enh,D15S11 enh)
CL08	GM11428	chr3	B-Lymphocyte	DUPLICATED CHROMOSOME	46,XX,der(3)dup(3q)inv(p26q22)
CL09	GM50178	chr5	B-Lymphocyte	CHROMOSOME DELETION	46,XX,del(5)(p15.3)pat.ish del(5)(p15.3)(C84C11/T3-,D5S23/D5S721+,EGR-1+,D5S2907+)pat
CL10	GM12959	chr1	B-Lymphocyte	CHROMOSOME DELETION	46,XY,del(1)(q43)
CL11	GM50275	chr5	B-Lymphocyte	CHROMOSOME DELETION	46,XY,del(5)(p14p14).ish del(5)(p14p14)(189N21+)

*CL04 was regarded as the negative sample because of its unbalanced translocation signal

Table II The summary of alignment situation of HiSeq2000

Sample	Class	Reads (M)	Length	Mapping Rate	Unique Reads	Mismatch Rate	Deletion Rate	Insertion Rate	Uniqe Mapping Rate	Coverage	Coverage of Depth	Duplication Rate
CL01	RM	2	29	79.32%	1192319	1.50%	0.05%	0.05%	59.62%	0.31%	3.84	0.96%
CL02	RM	2	29	79.45%	1178514	1.47%	0.04%	0.05%	58.93%	0.49%	2.46	0.99%
CL03	RM	2	29	77.21%	1145656	1.54%	0.05%	0.06%	57.28%	0.24%	4.9	1.13%
CL04	RM	2	29	76.40%	1158645	1.44%	0.05%	0.05%	57.93%	0.30%	3.87	0.99%
CL05	RM	2	29	79.22%	1215406	1.33%	0.04%	0.05%	60.77%	0.26%	4.72	0.95%
CL06	RM	2	29	77.58%	1176326	1.42%	0.04%	0.05%	58.82%	0.29%	4.18	1.07%
CL07	RM	2	29	78.54%	1177768	1.41%	0.04%	0.05%	58.89%	0.22%	5.51	1.11%
CL08	RM	2	29	77.63%	1184942	1.43%	0.05%	0.05%	59.25%	0.25%	4.85	1.07%
CL09	RM	2	29	78.87%	1195478	1.34%	0.04%	0.05%	59.77%	0.42%	2.91	1.04%
CL10	RM	2	29	77.59%	1170841	1.44%	0.05%	0.06%	58.54%	0.46%	2.6	1.03%
CL11	RM	2	29	78.16%	1174172	1.39%	0.04%	0.05%	58.71%	0.38%	3.16	1.10%
CL01	RS	2	29	68.37%	1044241	1.61%	0.05%	0.07%	52.21%	0.12%	8.6	3.31%
CL02	RS	2	29	77.49%	1193625	1.37%	0.04%	0.05%	59.68%	0.34%	3.54	0.75%
CL03	RS	2	29	78.51%	1184833	1.53%	0.05%	0.06%	59.24%	0.27%	4.47	0.91%
CL04	RS	2	29	78.54%	1206274	1.36%	0.05%	0.05%	60.31%	0.42%	2.93	0.96%
CL05	RS	2	29	76.65%	1182444	1.45%	0.05%	0.06%	59.12%	0.29%	4.09	0.94%
CL06	RS	2	29	79.07%	1226832	1.36%	0.04%	0.05%	61.34%	0.40%	3.12	0.92%
CL07	RS	2	29	70.56%	1064757	1.45%	0.05%	0.06%	53.24%	0.41%	2.6	1.14%
CL08	RS	2	29	78.09%	1204199	1.40%	0.04%	0.05%	60.21%	0.38%	3.26	0.89%
CL09	RS	2	29	78.51%	1192598	1.37%	0.05%	0.05%	59.63%	0.37%	3.25	0.95%
CL10	RS	2	29	77.07%	1181974	1.47%	0.05%	0.06%	59.10%	0.20%	6.02	1.03%
CL11	RS	2	29	77.79%	1181239	1.42%	0.04%	0.05%	59.06%	0.27%	4.51	1.13%
CL01	SM	2	29	85.20%	1280654	1.96%	0.03%	0.03%	64.03%	0.25%	5.11	1.80%
CL02	SM	2	29	84.30%	1266797	1.93%	0.03%	0.03%	63.34%	0.27%	4.69	2.14%
CL03	SM	2	29	83.11%	1203680	2.12%	0.04%	0.03%	60.18%	0.32%	3.79	2.00%
CL04	SM	2	29	82.89%	1218049	2.12%	0.04%	0.03%	60.90%	0.28%	4.35	1.79%
CL05	SM	2	29	83.78%	1238665	1.99%	0.04%	0.03%	61.93%	0.34%	3.67	1.63%
CL06	SM	2	29	83.10%	1174067	1.74%	0.03%	0.03%	58.70%	0.31%	3.88	1.85%
CL07	SM	2	29	84.52%	1280201	1.83%	0.03%	0.02%	64.01%	0.34%	3.78	2.77%
CL08	SM	2	29	84.14%	1275016	1.92%	0.03%	0.03%	63.75%	0.35%	3.67	2.73%
CL09	SM	2	29	84.10%	1222447	1.77%	0.03%	0.03%	61.12%	0.23%	5.28	1.90%
CL10	SM	2	29	84.61%	1209815	1.69%	0.03%	0.03%	60.49%	0.33%	3.7	1.80%
CL11	SM	2	29	82.55%	1237455	1.84%	0.03%	0.03%	61.87%	0.40%	3.16	2.29%
CL01	SS	2	29	81.83%	1259267	2.02%	0.03%	0.03%	62.96%	0.40%	3.22	6.04%
CL02	SS	2	29	73.91%	1165444	1.42%	0.02%	0.02%	58.27%	0.24%	4.95	2.62%
CL03	SS	2	29	83.43%	1262344	1.93%	0.03%	0.03%	63.12%	0.52%	2.45	2.97%
CL04	SS	2	29	80.55%	1225461	1.90%	0.04%	0.03%	61.27%	0.21%	5.98	5.65%
CL05	SS	2	29	84.14%	1284989	1.79%	0.04%	0.03%	64.25%	0.30%	4.34	3.56%
CL06	SS	2	29	84.57%	1285387	1.77%	0.03%	0.02%	64.27%	0.26%	5.04	4.67%
CL07	SS	2	29	82.61%	1257331	1.75%	0.03%	0.02%	62.87%	0.25%	5.16	7.85%
CL08	SS	2	29	85.03%	1300106	1.74%	0.03%	0.02%	65.01%	0.27%	4.86	4.76%
CL09	SS	2	29	83.31%	1266234	1.78%	0.03%	0.02%	63.31%	0.30%	4.25	8.68%
CL10	SS	2	29	85.04%	1299006	1.66%	0.03%	0.02%	64.95%	0.32%	4.13	7.90%
CL11	SS	2	29	82.50%	1257374	1.65%	0.03%	0.02%	62.87%	0.26%	4.96	4.00%

Table III The summary of alignment situation of Proton

Sample	Class	Reads (M)	Production(Mb)	Unique Reads	Unique Map Rate	Length Range	Mismatch Rate	Deletion Rate	Insertion Rate	Gap Rate	Coverage	Coverage OfDepth	Duplication
CL01	RM	2	234.22	1850944	92.56%	30-353	1.41%	0.36%	0.38%	0.74%	5.50%	1.4	9.34%
CL02	RM	2	237.83	1846104	92.32%	30-349	1.44%	0.38%	0.38%	0.77%	5.71%	1.37	7.47%
CL03	RM	2	236.3	1815073	90.82%	30-355	1.57%	0.36%	0.38%	0.75%	5.40%	1.42	9.88%
CL04	RM	2	233.01	1831817	91.65%	30-352	1.60%	0.35%	0.46%	0.81%	5.52%	1.38	10.21%
CL05	RM	2	232.75	1845752	92.29%	30-346	1.40%	0.34%	0.43%	0.77%	5.44%	1.41	11.21%
CL06	RM	2	234.76	1837414	91.84%	30-353	1.44%	0.37%	0.45%	0.82%	5.41%	1.42	11.18%
CL07	RM	2	233.99	1831730	91.58%	30-354	1.64%	0.40%	0.43%	0.82%	5.41%	1.42	10.97%
CL08	RM	2	233.4	1836415	91.83%	30-352	1.46%	0.39%	0.40%	0.79%	5.55%	1.38	9.40%
CL09	RM	2	234.5	1839317	92.05%	30-354	1.41%	0.36%	0.43%	0.79%	5.45%	1.41	11.38%
CL10	RM	2	236.11	1836450	91.81%	30-352	1.44%	0.35%	0.48%	0.83%	5.49%	1.41	10.81%
CL11	RM	2	231.03	1826419	91.38%	30-353	1.53%	0.37%	0.47%	0.84%	5.33%	1.42	11.00%
CL01	RS	2	225.54	1733874	86.72%	30-352	1.60%	0.33%	0.42%	0.74%	3.29%	2.12	11.34%
CL02	RS	2	241.72	1858194	92.92%	30-354	1.41%	0.35%	0.36%	0.72%	5.63%	1.42	8.96%
CL03	RS	2	238.59	1835991	91.87%	30-354	1.52%	0.36%	0.40%	0.77%	5.69%	1.37	7.46%
CL04	RS	2	238.53	1850111	92.46%	30-354	1.48%	0.36%	0.38%	0.73%	5.76%	1.36	8.46%
CL05	RS	2	228.8	1838071	91.94%	30-353	1.45%	0.33%	0.48%	0.80%	5.33%	1.41	11.50%
CL06	RS	2	236.09	1845947	92.32%	30-353	1.46%	0.37%	0.44%	0.81%	5.36%	1.45	12.45%
CL07	RS	2	239.61	1708914	85.45%	30-353	1.45%	0.38%	0.45%	0.83%	4.99%	1.47	14.00%
CL08	RS	2	234.83	1842085	92.14%	30-350	1.44%	0.36%	0.43%	0.79%	5.41%	1.43	11.53%
CL09	RS	2	227.29	1830172	91.49%	30-352	1.42%	0.31%	0.48%	0.79%	5.20%	1.43	12.43%
CL10	RS	2	232.43	1828760	91.44%	30-352	1.37%	0.36%	0.43%	0.79%	5.47%	1.39	10.07%
CL11	RS	2	235.91	1837734	91.84%	30-353	1.62%	0.38%	0.42%	0.80%	5.55%	1.39	10.23%
CL01	SM	2	211.05	1823185	91.17%	30-346	1.27%	0.28%	0.47%	0.76%	5.10%	1.35	10.76%
CL02	SM	2	220.97	1818568	90.90%	30-353	1.30%	0.32%	0.49%	0.80%	5.20%	1.38	10.84%
CL03	SM	2	215.62	1802054	90.11%	30-353	1.29%	0.33%	0.46%	0.80%	5.04%	1.38	12.21%
CL04	SM	2	210.29	1816554	90.79%	30-352	1.32%	0.31%	0.43%	0.75%	5.15%	1.32	10.88%
CL05	SM	2	213.05	1826736	91.33%	30-352	1.29%	0.30%	0.46%	0.75%	5.05%	1.37	13.04%
CL06	SM	2	211.69	1815563	90.75%	30-353	1.34%	0.30%	0.47%	0.77%	5.18%	1.32	10.91%
CL07	SM	2	221.38	1832555	91.65%	30-353	1.27%	0.33%	0.47%	0.79%	5.04%	1.44	12.88%
CL08	SM	2	223.45	1844279	92.15%	30-353	1.18%	0.26%	0.35%	0.61%	5.23%	1.4	12.54%
CL09	SM	1.92	211.61	1749051	90.98%	30-353	1.24%	0.28%	0.33%	0.61%	4.98%	1.38	13.51%
CL10	SM	2	222.27	1825469	91.27%	30-352	1.29%	0.27%	0.36%	0.63%	5.35%	1.35	11.29%
CL11	SM	2	227.62	1838189	91.91%	30-354	1.12%	0.28%	0.36%	0.64%	5.29%	1.41	12.84%
CL01	SS	2	217.54	1842068	92.07%	30-355	1.26%	0.31%	0.48%	0.79%	4.63%	1.54	12.52%
CL02	SS	2	222.21	1804517	90.25%	30-354	1.36%	0.33%	0.51%	0.83%	4.94%	1.45	10.98%
CL03	SS	2	215.09	1807415	90.38%	30-354	1.19%	0.32%	0.49%	0.80%	4.84%	1.43	11.95%
CL04	SS	2	218.02	1795544	89.79%	30-352	1.39%	0.34%	0.49%	0.83%	4.48%	1.56	12.75%
CL05	SS	2	211.41	1825169	91.28%	30-352	1.35%	0.30%	0.51%	0.81%	4.77%	1.44	12.33%
CL06	SS	2	219.45	1832564	91.62%	30-353	1.28%	0.35%	0.48%	0.83%	4.64%	1.55	13.65%
CL07	SS	2	219.64	1819845	91.01%	30-354	1.38%	0.31%	0.51%	0.83%	4.17%	1.71	15.15%
CL08	SS	2	227.08	1841986	92.12%	30-349	1.28%	0.28%	0.39%	0.66%	4.83%	1.55	13.64%
CL09	SS	2	226.37	1841177	92.04%	30-354	1.33%	0.28%	0.40%	0.68%	4.15%	1.79	15.38%
CL10	SS	2	229.66	1839407	91.96%	30-353	1.29%	0.30%	0.39%	0.69%	4.19%	1.8	17.41%
CL11	SS	2	226.69	1838385	91.90%	30-354	1.34%	0.30%	0.39%	0.69%	4.94%	1.5	11.95%

Table IV Detection results summary

Sample Name	Class	SNP array	Hiseq2000_Karyotype			Proton_Karyotype		
			CV	Status	False positive signals	CV	Status	False positive signals
CL01-RS	RS	46,X,del(X)(q13)	0.8138	Fail		0.7576	Fail	
CL02-RS	RS	46,XY, del(17)(q21.3q23)	0.3983	√	del(7q11.21-7q11.23)(14.74M) dup(13q14.11-13q14.3)(12.32M)	0.5135	×	dup(Xq21.1-Xq21.32)(15.37M) del(13q13.2-13q14.3)(27.62M) dup(Yp11.31-Yp11.2)(5.02M)
CL03-RS	RS	49,XYYYY	0.3724	√		0.5095	√	
CL04-RS	RS	46,XX,t(1;6)	0.3139	N		0.4787	N	
CL05-RS	RS	46,XX, del(2)(q34q36)	0.2961	√		0.4293	√	
CL06-RS	RS	46,dup(X)(q28),del(Y)(q11.2)	0.4264	×	del(15q11.2-15q13.3)(8.98M)	0.5946	×	
CL07-RS	RS	46,XY, dup(15)(q11q12)	0.3572	√		0.5909	√	dup(Yp11.31-Yq12)(26.16M) dup(Xq11.1-Xq21.1)(31.27M)
CL08-RS	RS	46,XX, dup(3q)(p26q22)	0.3793	√	del(9p13.2-9p11.2)(8.10M)	0.5212	√	
CL09-RS	RS	46,XX,del(5)(p15.3)	0.3092	√		0.4789	√	
CL10-RS	RS	46,XY,del(1)(q43)	0.3344	√		0.5619	√	dup(Yp11.31-Yq12)(26.16M) dup(Xq11.1-Xq21.1)(16.88M)
CL11-RS	RS	46,XY, del(5)(p14p14)	0.3429	√		0.5588	√	dup(Yp11.31-Yp11.2)(5.76M)
CL01-RM	RM	46,X,del(X)(q13)	0.3029	√		0.4424	√	
CL02-RM	RM	46,XY, del(17)(q21.3q23)	0.3276	√		0.4981	√	dup(Yp11.31-Yq12)(26.16M) dup(Xq11.1-Xq21.1)(13.93M)
CL03-RM	RM	49,XYYYY	0.3226	√		0.4774	√	dup(Xq11.1-Xq21.32)(39.81M)
CL04-RM	RM	46,XX,t(1;6)	0.2968	N		0.4819	N	
CL05-RM	RM	46,XX, del(2)(q34q36)	0.2913	√		0.4347	√	

CL06-RM	RM	46,dup(X)(q28),del(Y)(q11.2)	0.323	×		0.5187	×	
CL07-RM	RM	46,XY, dup(15)(q11q12)	0.3354	×		0.5464	√	dup(Xq11.1-Xq21.1)(14.93M)
CL08-RM	RM	46,XX, dup(3q)(p26q22)	0.3121	√		0.4861	√	
CL09-RM	RM	46,XX,del(5)(p15.3)	0.3	√		0.4932	√	
CL10-RM	RM	46,XY,del(1)(q43)	0.3186	√		0.5248	√	dup(Xq11.1-Xq21.1)(16.88M)
CL11-RM	RM	46,XY, del(5)(p14p14)	0.3255	√		0.544	√	dup(Yp11.31-Yq12)(26.16M)
CL01-SS	SS	46,X,del(X)(q13)	0.3903	√		0.4957	√	
CL02-SS	SS	46,XY, del(17)(q21.3q23)	0.4988	√	del(7q11.21-7q11.22)(9.86M)	0.6546	√	
CL03-SS	SS	49,XYYYY	0.3027	√		0.494	√	
CL04-SS	SS	46,XX,t(1;6)	0.4431	N		0.4379	N	
CL05-SS	SS	46,XX, del(2)(q34q36)	0.3076	√		0.4624	√	
CL06-SS	SS	46,dup(X)(q28),del(Y)(q11.2)	0.3711	×	del(7q11.21-7q11.23)(14.24M) dup(11q11-11q14.1)(25.20M)	0.5217	×	dup(11q11-11q14.1)(27.81M)
CL07-SS	SS	46,XY, dup(15)(q11q12)	0.469	×	dup(Xq11.1-Xq21.1)(15.22M)	0.568	×	dup(Xq11.1-Xq21.1)(18.22M)
CL08-SS	SS	46,XX, dup(3q)(p26q22)	0.3741	√		0.4542	√	dup(9q12-9q21.11)(6.41M)
CL09-SS	SS	46,XX,del(5)(p15.3)	0.4279	×		0.5149	√	del(9p13.2-9p11.2)(8.54M)
CL10-SS	SS	46,XY,del(1)(q43)	0.4179	√		0.5377	√	dup(Xq13.2-Xq22.1)(27.14M)
CL11-SS	SS	46,XY, del(5)(p14p14)	0.3485	√		0.4909	√	dup(Yp11.31-Yp11.2)(5.72M) dup(Xq11.1-Xq21.1)(15.95M)
CL01-SM	SM	46,X,del(X)(q13)	0.271	√		0.4435	√	

CL02-SM	SM	46,XY, del(17)(q21.3q23)	0.2973	√		0.4851	√	
CL03-SM	SM	49,XYYYY	0.279	√		0.4778	√	
CL04-SM	SM	46,XX,t(1;6)	0.2672	N		0.4379	N	
CL05-SM	SM	46,XX, del(2)(q34q36)	0.2804	√		0.4111	√	
CL06-SM	SM	46,dup(X)(q28),del(Y)(q11.2)	0.299	×	dup(Xq11.1-Xq21.1)(21.27M)	0.4385	×	dup(Xq11.2-Xq21.1)(17.14M)
CL07-SM	SM	46,XY, dup(15)(q11q12)	0.3144	×	dup(9q12-9q21.11)(5.89M)	0.4736	×	dup(Xp11.22-Xp11.21)(5.99M) dup(Xq11.1-Xq21.1)(16.69M)
CL08-SM	SM	46,XX, dup(3q)(p26q22)	0.2774	√		0.4416	√	
CL09-SM	SM	46,XX,del(5)(p15.3)	0.27	√		0.4256	√	dup(9q12-9q21.11)(5.87M)
CL10-SM	SM	46,XY,del(1)(q43)	0.2973	√	dup(Xq11.1-Xq21.33)(34.28M) dup(Xq23-Xq25)(12.63M)	0.4353	√	dup(Xp11.3-Xp11.21)(12.55M)
CL11-SM	SM	46,XY, del(5)(p14p14)	0.2935	√		0.4882	√	dup(Xq11.1-Xq21.1)(37.91)

"√" represents that the detection results are consistent with the standard results

"X" represents that detection failed

"Fail" represents that sample quality failed

"N" represents the negative sample

Table V All the detection results for two platforms

Combinations	Index	HiSeq2000	Proton
RM	FNR	2/11	1/11
RM	TNR	1/1	1/1
RM	FPR	0/11	6/11
RM	TPR	8/11	9/11
RS	FNR	1/10	2/10
RS	TNR	1/1	1/1
RS	FPR	4/10	8/10
RS	TPR	8/10	7/10
SS	FNR	3/11	2/11
SS	TNR	1/1	1/1
SS	FPR	4/11	7/11
SS	TPR	7/11	8/11
SM	FNR	2/11	2/11
SM	TNR	1/1	1/1
SM	FPR	4/11	6/11
SM	TPR	8/11	8/11
